# Late-onset metastatic adenocarcinoma of the spermatic cord from primary gastric cancer

**DOI:** 10.1186/1477-7819-12-128

**Published:** 2014-04-27

**Authors:** Jae Heon Kim, Doo Sang Kim, Hyun Deuk Cho, Moon Su Lee

**Affiliations:** 1 Department of Urology, Soonchunhyang University Hospital, Soonchunhyang University College of Medicine, Seoul, Korea; 2 Department of Urology, Soonchunhyang University Cheonan Hospital, Soonchunhyang University College of Medicine, 23-20, Bongmyeongdong, Cheonan, Chungnam 330-721, Korea; 3 Department of Pathology, Soonchunhyang University Cheonan Hospital, Soonchunhyang University College of Medicine, Cheonan, Korea; 4 Department of Surgery, Soonchunhyang University Cheonan Hospital, Soonchunhyang University College of Medicine, Cheonan, Korea

**Keywords:** Metastasis, Spermatic cord, Epididymis, Adenocarcinoma, Gastric cancer

## Abstract

**Background:**

Metastatic cancers of the paratesticular tissue are very rare; however, the most frequent primary site of spermatic cord metastasis is the gastrointestinal tract.

**Case presentation:**

We recently observed two cases of late-onset metastatic adenocarcinoma of the spermatic cord. Both patients complained of groin discomfort with a palpable mass in the scrotum and inguinal area. Radical orchiectomy and adjuvant chemotherapy were performed in both patients. Although the prognosis of patients with metastatic adenocarcinoma of the spermatic cord is typically poor, the prognosis of our patients was favorable after follow-up for 14 to 18 months.

**Conclusions:**

In patients with groin discomfort or swelling and a history of gastric cancer, metastatic adenocarcinoma should be included in the differential diagnosis for early detection of tumors.

## Background

Gastric cancer is the second most common cause of cancer-related deaths worldwide. In Korea, gastric cancer is the most common cancer, affecting over 25,000 individuals annually [1]. Sites of recurrence of gastric cancer include the peritoneum, liver, bone marrow, lymph nodes, and, very rarely, the paratesticular tissues including the spermatic cord. The prognosis of a metastatic tumor of the spermatic cord is unfavorable, as those tumors are usually detected in the setting of disseminated diseases [2]. Herein, we report two patients with gastric cancer recurrence in the spermatic cord that occurred 6 to 7 years after total and subtotal gastrectomy and adjuvant chemotherapy with favorable prognoses.

## Case presentation

### Case 1

A 49-year-old man was referred to the Outpatient Department at Soonchunhyang University Cheonan Hospital (Cheonan, Korea) with a palpable mass in his right scrotum and discomfort in the spermatic cord persisting for 2 months. The mass was characterized with an insidious onset; it gradually increased in size. Seven years previously, the patient had undergone subtotal gastrectomy with gastrojejunostomy, segmental resection of the transverse colon, and end-to-end anastomosis for advanced gastric cancer. Histologic examination of the specimen revealed mucinous adenocarcinoma with signet ring cell carcinoma (T3N2M0, stage IV). The patient had received adjuvant chemotherapy with six cycles of 5-fluorouracil and cisplatin. The patient had been followed-up for 7 years on an outpatient basis, and he had not shown any sign of recurrence.

**Figure 1:**
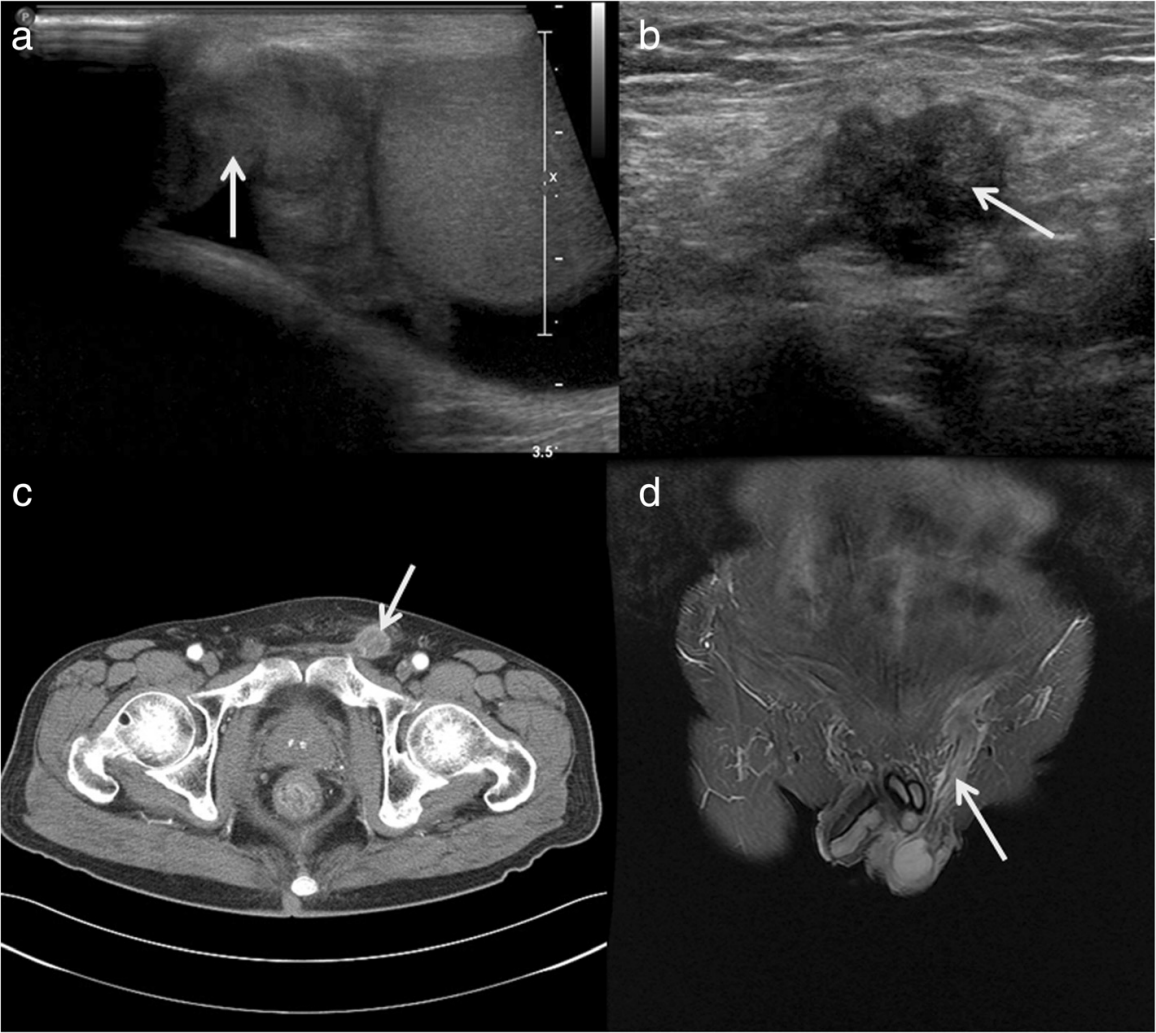
**Ultrasonography, CT, and MRI. (a)** Case 1: scrotal ultrasonography revealed an ill-defined heterogeneous mass (white arrow) in the spermatic cord and epididymis. **(b)** Case 2: inguinal ultrasonography revealed an ill-defined heterogeneous mass (white arrow) of the spermatic cord in the inguinal area, and **(c,d)** CT and MRI revealed an ill-defined enhancing mass (white arrows), longitudinally along the inguinal tract. CT, computed tomography; MRI, magnetic resonance imaging.

On physical examination, a 3- × 2-cm mass was palpated above the right testis. The mass was hard, nodular, and non-tender. Scrotal ultrasonography revealed a 3- × 2-cm-sized heterogeneous mass with irregular contours in the right epididymal head, and it extended into the spermatic cord (Figure 1a).

**Figure 2:**
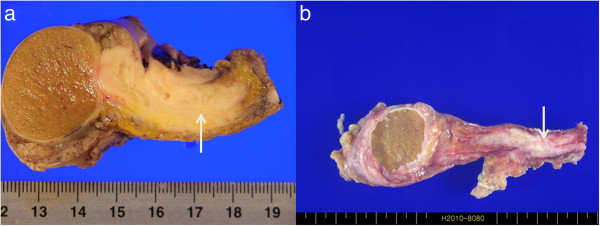
**Gross examination.** Gross examination showed an ill-defined grayish-white solid mass (white arrows) in the epididymis and spermatic cord. **(a)** Case 1. **(b)** Case 2.

**Figure 3:**
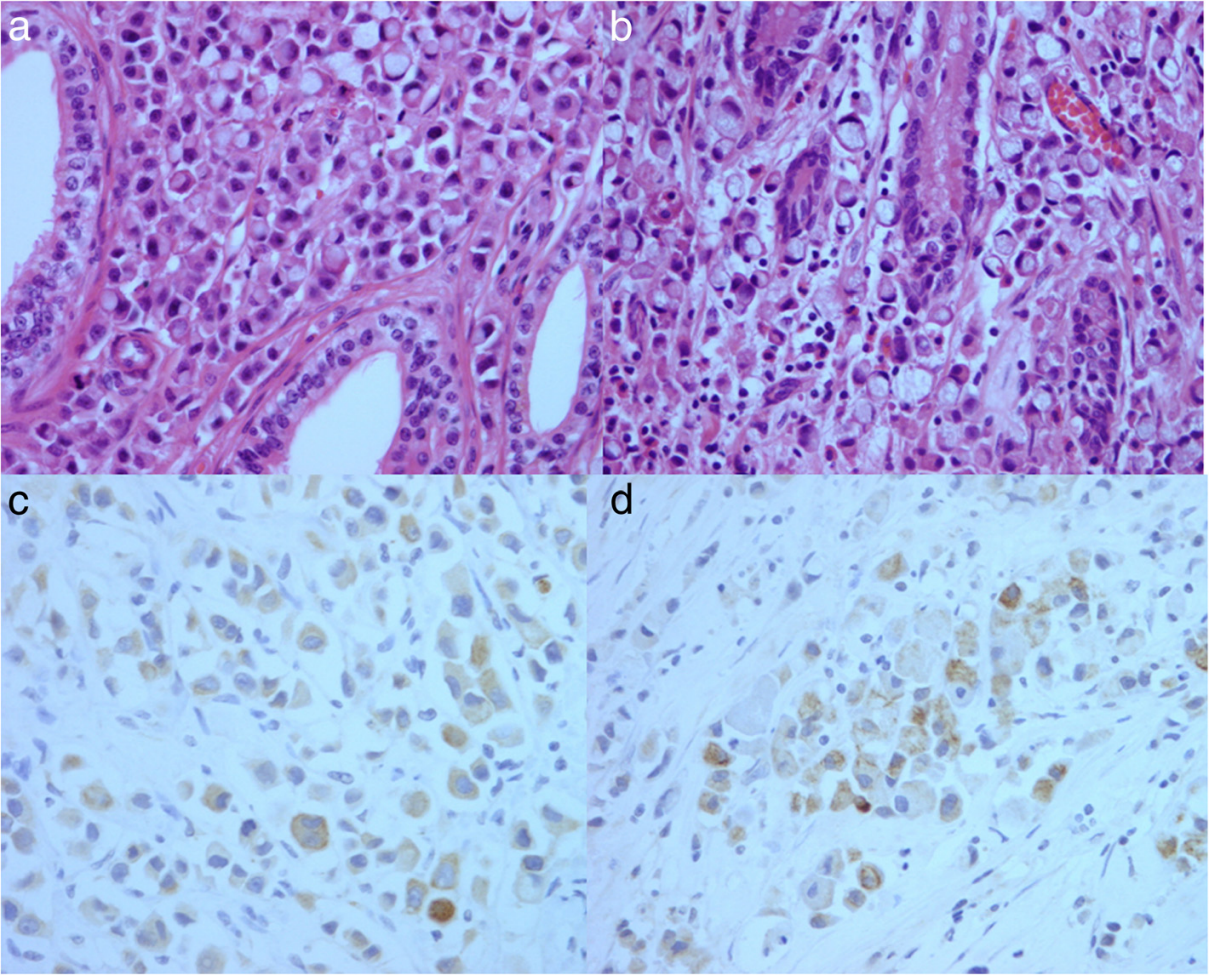
**Histological analysis. Histological analysis revealed an adenocarcinoma with signet ring cells. (a)** Case 1: epididymal tubules. **(b)** Case 2: stomach (H & E staining, 400×). Immunohistochemical staining revealed a cytokeratin 7-positive mass. **(c)** Case 1: epididymal tubules. **(d)** Case 2: stomach (400×). H & E, hematoxylin and eosin.

The patient underwent transinguinal exploration. Frozen biopsy revealed a mucinous carcinoma of the epididymis. The specimen was removed via radical inguinal orchiectomy. Grossly, the specimen was an ill-defined grayish-white solid mass (4.0 × 1.5 × 1.5 cm) in the epididymis and spermatic cord (Figure 2a). Histopathological examination revealed adenocarcinoma with signet ring cells in the epididymis, spermatic cord, and soft tissue (Figure 3a). The results of immunohistochemical staining were focally positive for cytokeratin 7/20 and negative for prostate specific antigen (PSA), consistent with previous findings of gastric cancer.

We performed systemic reevaluation including gastroduodenoscopy, total colonoscopy, abdominal computed tomography (CT), and total-body positron emission tomography (PET), none of which could confirm another primary tumor. The levels of tumor markers such as PSA, carcinoembryonic antigen (CEA), beta-human chorionic gonadotrophin (β-HCG), alpha-fetoprotein (AFP), carbohydrate antigen 19–9 (CA19-9), and cancer antigen 125 (CA125) were normal. The patient received eight courses of adjuvant chemotherapy with the FOLFOX regimen (folinic acid, fluorouracil, oxaliplatin). He has been doing well with no evidence of recurrence after radical orchiectomy at 26 months of follow-up.

### Case 2

A 60-year-old man presented to the Urology Department at Soonchunhyang University Seoul Hospital with left inguinal pain for 1 month. The patient had a history of T3M3M0 gastric adenocarcinoma and moderate differentiation 6 years previously. He had been treated with total gastrectomy followed by three cycles of capecitabine and cisplatin chemotherapy. After chemotherapy, the patient had no evidence of recurrence of cancer at the 6-year follow-up. Results of laboratory tests including the levels of the tumor markers, including PSA, CEA, CA19-9, β-HCG, AFP, and CA125, were within the normal range. Physical examination revealed moderate right scrotal tenderness and inguinal tenderness. Chest radiography did not reveal anything abnormal; the results of hematological and biochemical tests were within normal limits. Ultrasonography, pelvic CT, and magnetic resonance imaging (MRI) revealed a soft tissue mass with irregular margins in the spermatic cord (Figure 1b,c,d). The patient underwent radical orchiectomy via an inguinal approach. Gross findings revealed an ill-defined grayish-white solid mass (2.0 × 3.5 × 1.5 cm) in the spermatic cord (Figure 2b). Pathological analysis revealed mucinous adenocarcinoma with moderate differentiation (Figure 3b). All resection margins were free of carcinoma. Immunohistochemical staining for D2-40 did not reveal any lymphatic invasion. After the patient recovered from radical orchiectomy, colonoscopic biopsy was performed for rectal wall thickening, and it revealed adenocarcinoma with moderate differentiation. Ten cycles of adjuvant radiation therapy were administered. At the 20-month follow-up, the patient was stable and demonstrated no recurrence of cancer.

## Discussion

Metastatic tumors of the spermatic cord are rare [3]. The most common primary site of spermatic cord metastasis is the gastrointestinal tract, followed by the pancreas, prostate, and kidneys [4]. The mechanisms involved in metastasis to the spermatic cord and epididymis from primary malignant neoplasms are unclear. However, several routes have been proposed [3]. The main routes are the hematogenous or lymphatic routes, but other routes including retrograde extension through the vessel, either along its lumen or by direct extension via the wall of the vessel, and transperitoneal seeding through the patent tunica vaginalis have been proposed [3,4]. In the cases we have discussed here, the possibility of hematogenous or lymphatic spread cannot be excluded. In both cases, a hydrocele was noted, which indicates that lymphatic drainage could be impaired, leading to congestion.

Moon *et al*. [5] have recently suggested that tumor factors, including the stage, were clinical prognostic indicators within 5 years post-gastrectomy, but there were no such indicators after 5 years.

In patients with advanced gastric carcinoma undergoing radical gastrectomy and adjuvant chemotherapy, approximately half the patients survive for > 5 years [5]. After gastrectomy, 8.8% of patients relapse with the predominant pattern of distant metastasis (34.6%) during the 5 to 10 years after surgery. However, recurrences many years after treatment may be related to the activation of long-lasting tumor dormancy, particularly in distant organs [5].

The prognosis of a metastatic tumor of the epididymis and spermatic cord is typically unfavorable [2], as metastatic tumors to the paratesticular tissues are usually detected in the setting of disseminated diseases. However, previous reports of metastatic tumors of the epididymis and spermatic cord from gastric cancer were published in 1980 to 1990. During the last two decades, chemotherapy including adjuvant chemotherapy has evolved significantly in both its efficacy and toxicity. The main reasons that our patients had favorable outcomes were owing to the relatively early detection because of the development of radiologic imaging, and also owing to the effect of adjuvant chemotherapy. Earlier reports might not have considered the positive effect of adjuvant chemotherapy. Considering these diseases in the differential diagnosis of a groin mass when a history of gastrointestinal tract cancer is present can be critical for a more precise diagnosis and more targeted treatment.

## Conclusion

Metastatic lesions of the spermatic cord should be included in the differential diagnosis of a scrotal or inguinal mass with discomfort, especially in patients with a history of gastrointestinal cancer, to promptly achieve a targeted treatment.

## Consent

Written informed consent was obtained from the patients for publication of these case reports and any accompanying images.

## Abbreviations

AFP: Alpha-fetoprotein; CA125: Cancer antigen 125; CA19-9: Carbohydrate antigen 19–9; CEA: Carcinoembryonic antigen; CT: Computed tomography; H & E: Hematoxylin and eosin; MRI: Magnetic resonance imaging; PET: Positron emission tomography; PSA: Prostate specific antigen; β-HCG: Beta-human chorionic gonadotrophin.

## Competing interests

The authors declare that they have no competing interests.

## Authors’ contributions

JHK and DSK wrote the paper, HDC carried out the pathologic confirmation, MSL have designed the concept and supervised. All authors read and approved the final manuscript.
